# Antonello Covacci (1957–2023), a visionary and beautiful mind

**DOI:** 10.1038/s44318-024-00167-5

**Published:** 2024-07-15

**Authors:** Rino Rappuoli, Duccio Medini

**Affiliations:** 1 Fondazione Biotecnopolo di Siena, Siena, Italy; 2 Fondazione Toscana Life Sciences, Siena, Italy

## Abstract

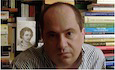

The EMBO member Antonello Covacci was born on 14 December 1957 in Tuscania, a town near Rome that, in antiquity, played a leading role in the Etruscan world. His father was of Istrian origin, had escaped the Foibe massacres that followed the Second World War, and died when Antonello was 22 years old. He was the principal of a secondary school and left his son a passion for Latin and classical studies. Antonello’s mother was a constant presence in her son’s discourses if he considered you a friend. At the end of his regular schooling in classic studies in 1976, Antonello transferred to Siena with his future wife and architect Carla. He started working at the Sclavo Research Center in 1983, then he graduated in medicine at the University of Florence in 1988.
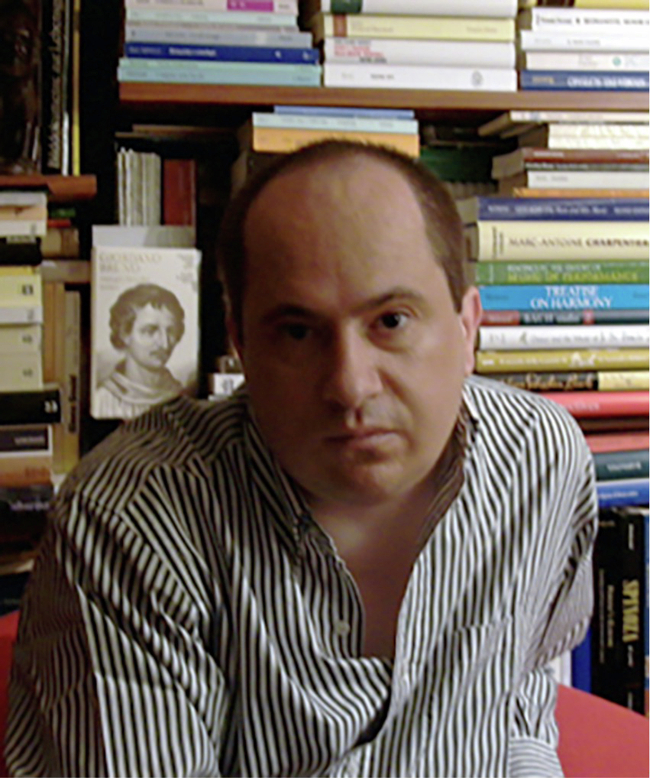


Antonello’s love for science was easy to spot. He had a renaissance view of innovation; he was interested in any aspect of science and was a truly visionary person with the capability of seeing what lay many years ahead. His desk and his house were full of copies of scientific papers, disposed in a beautiful, clean, and searchable order. Our laboratory at that time was dealing with making a vaccine against pertussis and studying the virulence of *Helicobacter pylori*, a bacterium that Barry Marshall and Robin Warren had just shown to cause peptic ulcers and gastric cancer. Antonello decided to focus on *Helicobacter* to discover factors responsible for virulence and cancer. Very early, he identified a Cytotoxin Associated Gene, which codes for an immunodominant antigen (CagA) present in all isolates associated with duodenal ulcers. CagA became his passion; he started to collaborate with Stanley Falkow from Stanford University and spent time in his lab. Out of this intense collaboration, they discovered that the CagA antigen is encoded by a genomic island composed of 31 genes, which encodes for a type IV secretion machinery that delivers CagA into the epithelial cells. They showed that, once inside the host cells, CagA is phosphorylated by a cellular kinase and then starts to disrupt the eukaryotic signal transduction pathways, inducing cytoskeletal plasticity and alteration of the tight junctions of the epithelium, slowly starting the process that eventually leads to disease and cancer. Still today, CagA is the only bacterial protein linked to cancer. Antonello was also fascinated by the genetics of *Helicobacter*, a bacterium that is transmitted vertically, mostly from mother to child. Connecting across disciplines the relationships between bacteria and humans, he realized that the pathogen co-evolved with humans. This allowed him to map the genetic geography of the pathogen *Helicobacter pylori* through its co-evolution with human migrations in history.

In the meantime, genome sequencing was making progress and Antonello, who had a strong passion for databases and computers, took the position to lead the biocomputing group of the Chiron Research Center in Siena. Shortly after, we started a collaboration with The Institute for Genomic Research (TIGR) founded by Craig Venter to sequence the genome of meningococcus B. Our need for sequence analysis skyrocketed. We gave Antonello the task of hiring people who could help organize and mine the genomes. He came back, proposing to recruit people with a degree in physics who had never done biology work and to put them in the same room with our bioinformaticians. It was a great decision, and the work that our bioinformatics group used to do in 6 months started to be done in 4 months, 2 months, 1 month, and ultimately 1 week. He also realized that the computing power we had was not enough for our needs. So, he decided to build for our small research center one of the first examples of grid computing systems applied to biology. By integrating multiple architectures behind a common middleware, the computational resources were made available transparently to the users. Simple and very complex tasks could be performed easily on the same system. Antonello was not directly involved in the use of genomics to discover the vaccine antigens that eventually led to the development of the meningococcus B vaccine, but his team provided the computing infrastructure and competence to make possible the development of the 4CMenB vaccine by the process named “reverse vaccinology”.

The collaboration with TIGR continued, and a few years later, we were the first group to have the genomic sequence of six different isolates of bacteria belonging to the same species, serotype B *Streptococcus*. Surprisingly, the computer analysis of the six genomes and of two additional genomes that became available in the meantime showed that each isolate had unique genes that were not present in any of the other isolates. The unexpected observation generated an animated discussion that lasted several months until the day when Antonello came up with the idea to call “pangenome” the collection of all the genes present in any member of the species while each member would have its own genome The idea was brilliant, and we agreed immediately to adopt the new word and the new way to classify genes. Today, the concept of the pangenome (or pan-genome), which includes all genes present in one species, has been described in more than 32,000 papers and is being used for every species, including humans.

Antonello was a visionary and this often made his life difficult: he could see things before any person could even start to imagine them, but it was difficult for him to communicate what he had in mind. We used to imagine him on top of a mountain, seeing on the other side a world that did not exist on our side, and he would describe it assuming that everybody was observing what he was seeing. Most of the people, including us, often did not understand what he was talking about. However, those times that we were able to capture his vision were transformative. In other cases, only now we are starting to understand what he had in mind more than 20 years ago. At some point in the late 90s, he presented a project he codenamed “Pico” after the Italian renaissance humanist Pico della Mirandola. He was describing databases containing all the scientific literature that could be searched intelligently to identify relationships and extract meaning. The database would contain all written documents, all data generated, so that everything could be searched and used by what we call today Artificial Intelligence. We remember him making a diagram with the structure of this project, and we recognize now that what he was describing at that time is not so different from today’s ChatGPT.

An unsatiable consumer of knowledge, Antonello would await anxiously every week the printed journals to digest avidly their content. Generous as he was, he always made available that encyclopedic knowledge to anyone who would ask him. He would get the most pleasure from spending time with young scientists seeking his advice to navigate interdisciplinary connections across biology, medicine, computing, literature, fine arts, and renaissance music. His conversations were always about science, and about what science could do for people. He did not have patience for low-quality science, and he would never compromise. He admired great scientists and enjoyed meeting Stanley Falkow, Craig Venter, Joerg Hacker, or spending time at Falkow’s Lab in Stanford.

He was very generous with others, but often, he forgot to seek help for himself when he may have needed it. The absolute beauty of a Da Victoria’s motet, the depth of Giordano Bruno’s reflections, the passion for a science that could help mankind, all in a pure, beautiful mind and a good, generous heart: that was Antonello Covacci. Science will miss his beautiful mind.

